# Supplementary material for: The genome sequence of Penthophera morio (Linnaeus, 1767) (Lepidoptera: Erebidae)
Source: Wellcome Open Res. 2026 Sep 11;11:626. [Version 1] doi: 10.12688/wellcomeopenres.27770.1 (PMC13658994; doi:10.12688/wellcomeopenres.27770.1)
Supplement: Peer review history

# Reviewer report: Reviewer response for version 1

**DOI:** 10.21956/wellcomeopenres.30561.r171632

**Published:** 2026-10-12

**Authors:** Ranjit Kumar Sahoo

**Affiliations:** 1 CSIR-Centre for Cellular and Molecular Biology (CCMB), Hyderabad, Telengana, India

In this Genome Note, the authors produced a genome assembly of *Penthophera. morio* based on the standardized methodology and analysis pipeline of ToL program. The paper clearly describes the accessibility of samples, sequences and methods. Sequence analysis has been conducted thoroughly to produce a reference quality genome assembly of the study species. This is a good addition to the genomic repository of butterflies and moths.

Are the datasets clearly presented in a usable and accessible format, and the assembly and annotation available in an appropriate subject-specific repository?

Yes

Are sufficient details of the sequencing and extraction, software used, and materials provided to allow replication by others?

Yes

Are the rationale for sequencing the genome and the species significance clearly described?

Yes

Are the protocols appropriate and is the work technically sound?

Yes

Reviewer Expertise:

Molecular Evolution, Genomics, Symbiosis

I confirm that I have read this submission and believe that I have an appropriate level of expertise to confirm that it is of an acceptable scientific standard.

# Reviewer report: Reviewer response for version 1

**Published:** 2026-09-11

**Authors:** Automated Benchmarking

**Affiliations:** 1 Wellcome Sanger Institute, Hinxton, UK

As this article is a Genome Note, Automated Benchmarking has been used to determine whether the reported assembly passes three defined checks established by the Earth BioGenome Project: the raw and assembled data are publicly available, the Earth BioGenome Project three-part metric is met, and the BUSCO score passes the stated threshold. For Penthophera morio (ilPenMori1), all three checks passed. Data available: pass. ENA study PRJEB102919, raw-read accessions ERR15896226 and ERR15896141, and assembly accessions GCA_977072185.1 and GCA_977072115.1 are public. Earth BioGenome Project three-part metric: pass. Haplotype 1 is reported as 7.C.Q67, meeting the 6.C.Q40 reference standard. BUSCO: pass. Haplotype 1, which is proposed as the reference assembly for this species, has 99.6% complete BUSCOs (99.1% single-copy and 0.5% duplicated). It meets the target of greater than 90% complete and single-copy BUSCOs. This decision uses values reported in the Genome Note and public accession records.

Reviewer Expertise:

NA

I confirm that I have read this submission and believe that I have an appropriate level of expertise to confirm that it is of an acceptable scientific standard.
